# Association of monocyte-lymphocyte ratio and myocardial infarction in the U.S. population with diabetes

**DOI:** 10.3389/fcvm.2024.1432838

**Published:** 2024-09-27

**Authors:** Yue Wu, Hong-Ju Xiang, Min Yuan

**Affiliations:** ^1^ Department of Cardiovascular Medicine, People’s Hospital of Xiangxi Tujia and Miao Autonomous Prefecture, The First Affiliated Hospital of Jishou University, Jishou, China; ^2^ Department of Neurology, Jiangxi Provincial People’s Hospital, The First Affiliated Hospital of Nanchang Medical College, Nanchang, China; ^3^ Department of Neurology, Xiangya Hospital, Central South University, Jiangxi Hospital, National Regional Center for Neurological Diseases, Nanchang, Jiangxi, China

**Keywords:** myocardial infarction, monocyte-lymphocyte ratio, diabetes, NHANES, cross-sectional

## Abstract

**Background and objective:**

The monocyte-to-lymphocyte ratio (MLR) has emerged as a novel inflammatory biomarker; however, its relationship with myocardial infarction (MI) in diabetic populations remains unclear. This study aimed to elucidate the association between MLR and MI prevalence in this unique population.

**Methods:**

This cross-sectional study used data from the National Health and Nutrition Examination Survey (NHANES), 2015-2018. MLR was utilized as both a continuous and categorical factor to examine its correlation with MI in individuals diagnosed with DM. Subgroup and sensitivity analyses were also performed.

**Results:**

In this study, 1,295 individuals with DM were enrolled, among whom 148 (11.4%) were diagnosed with MI. Patients with MI showed a greater MLR. Using a smoothed curve-fitting analysis, a linear relationship was observed between MLR and MI (p_for non−linearity_ = 0.27). Multivariate logistic regression analysis showed that MLR * 10 was positively correlated with the risk of MI (OR = 1.14, 95% CI 1.01∼1.29, *p* = 0.041). Compared with the lowest quartile, the OR for Q2, Q3, and Q4 were 2.13 (95% CI: 1.01∼4.47), 2.95 (95% CI: 1.45∼6.00), and 2.74 (95% CI: 1.32∼5.69), respectively. Subgroup analyses showed no significant interaction for MLR in any subgroup (all *P* > 0.05). The receiver operating characteristic (ROC) curve indicated that the area under the curve (AUCs) of MLR for predicting MI was 0.661 (95% CI: 0.617–0.706; *P* < 0.05).

**Conclusions:**

Our study demonstrated that MLR is significantly correlated with MI in patients with DM.

## Introduction

In the past few decades, cardiovascular disease (CVD) has become the primary worldwide reason for death. Myocardial infarction (MI), a severe cardiovascular condition with high mortality rates and bleak prognosis, has been a significant contributor to this trend. Over the years, revascularization therapies, primarily percutaneous coronary intervention (PCI) and coronary artery bypass grafting (CABG), have made substantial efforts to reduce MI-related mortality rates (1). However, the prevalence of MI has continued to rise, presenting itself as a pressing global public health challenge, with approximately seven million patients being diagnosed with MI annually (2, 3).

Diabetes mellitus (DM) is a chronic inflammatory condition characterized by increased insulin resistance and disrupted glucose regulation. The chronic hyperglycemic state in diabetes is associated with a cascade of inflammatory responses, which are now recognized as integral to the pathogenesis of the disease (4). Inflammation is thought to contribute to insulin resistance, *β*-cell dysfunction, and the development of vascular complications, which are the hallmarks of diabetes-associated morbidity and mortality (5).

Myocardial infarction (MI), a severe cardiovascular event, is significantly more common in individuals with diabetes (6). In patients with MI, irreversible damage to the heart tissue (infarction) due to severe ischemia of the heart muscle (7). The early stages of atherosclerosis involve an inflammatory response, and as atherosclerosis advances, this inflammation can contribute to various cardiovascular diseases including stroke and MI (8). Earlier studies have emphasized the crucial role of inflammation in the onset and development of MI (9).In the context of MI, inflammation is a double-edged sword. On the one hand, the body initiates a controlled inflammatory response to facilitate self-repair of damaged myocardial tissue. On the other hand, prolonged and excessive inflammation can exacerbate myocardial cell apoptosis and potentially lead to severe adverse events, negatively impacting patient outcomes (10, 11).

Monocytes are a type of white blood cell, crucial components of the immune system, playing a significant role in maintaining immune homeostasis and responding to infections and inflammatory reactions (12). Similarly, lymphocytes significantly contribute to immune responses, and heightened immune activation can reduce lymphocyte counts (13). Various inflammatory biomarkers, including white blood cells, neutrophils, lymphocytes, monocytes, platelets, and CRP, are commonly assessed in routine clinical practice. Combining these inflammatory biomarkers is expected to offer improved reproducibility and accuracy compared with a single biomarker. MLR is an emerging inflammatory biomarker that holds significance in predicting and prognosticating inflammation-related diseases such as cancer, cardiovascular diseases, and COVID-19 (14, 15). However, to our knowledge, the relationship between MLR and MI has not been studied. Hence, the main aim of this cross-sectional study was to investigate whether there is a connection between MLR and MI in adults with DM in the United States.

## Methods

### Study population

The National Health and Nutrition Examination Survey (NHANES) is conducted by the National Center for Health Statistics (NCHS) to evaluate the nutritional and health status of the US population, but not of institutions. The survey covered extensive evaluations, including physical and laboratory tests, along with inquiries about demographic, socioeconomic, and health factors. Approval for the project was granted by the Ethics Review Board of the National Center for Health Statistics and Research. As the data were available to the public, additional ethical authorization was not necessary. NHANES gathers demographic and health-related data through home visits, screenings, and laboratory tests in Mobile Examination Centers (MECs). Additional information regarding these datasets is available from the NHANES website (www.cdc.gov/nchs/nhanes/).

**Figure 1:**
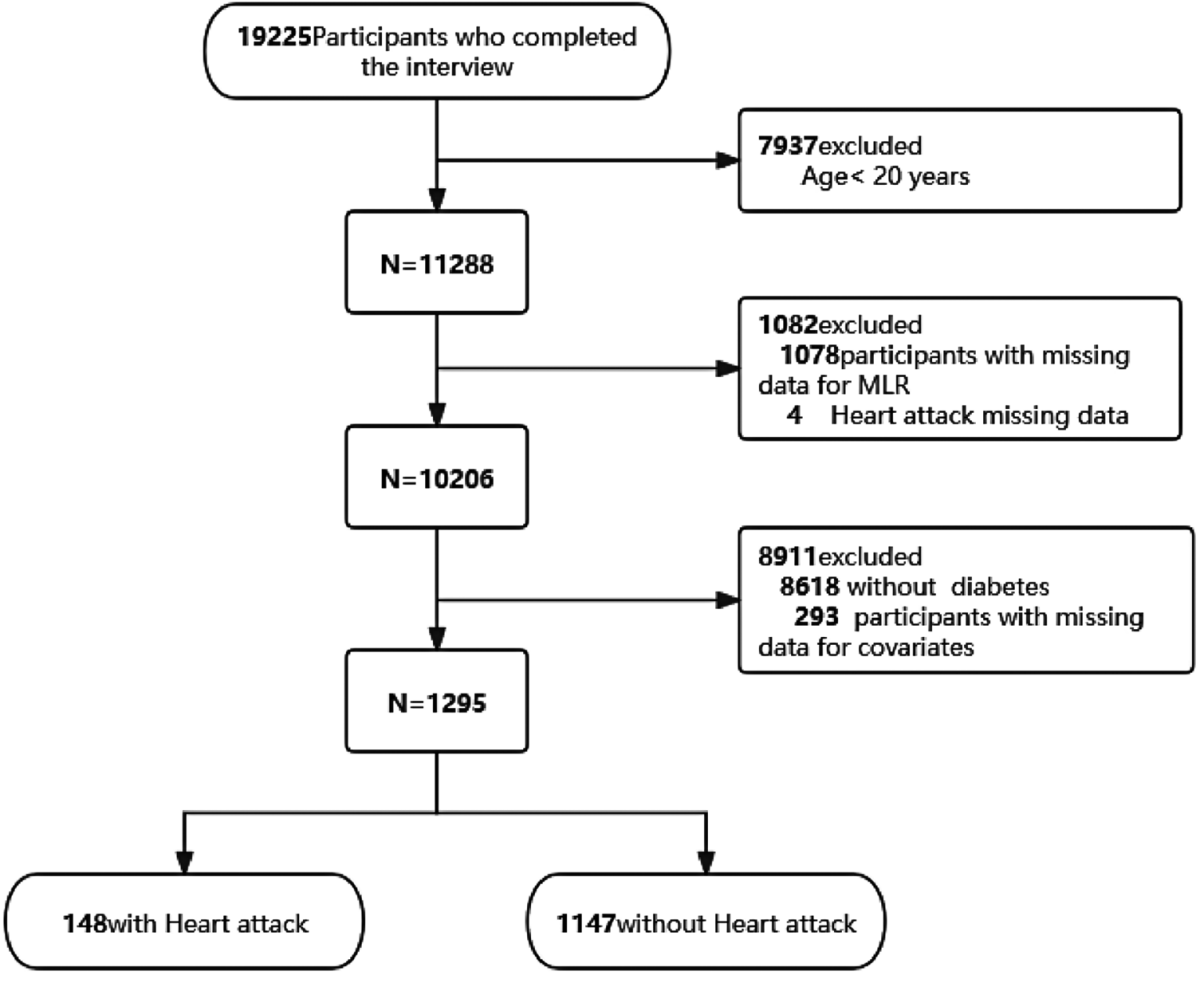
Flow chart of the study population inclusion.

We utilized two NHANES data cycles, covering 2015–2016 and 2017–2018. This analysis was conducted exclusively on adult participants aged 20 years and older. Initially, individuals lacking serum monocytes or lymphocytes (*n* = 1,078) were excluded. Subsequently, 4 individuals were excluded because of a lack of data related to MI, resulting in a refined sample size of 10,206 for further analysis. Participants who were non-diabetic (*n* = 8,618) and those with incomplete data on covariates (*n* = 293) were then further excluded (Figure 1). Ultimately, a total of 1,295 individuals were included in the analysis.This study followed the recommendations provided by the Strengthening the Reporting of Observational Studies in Epidemiology (STROBE) guidelines.

### Study variables and outcome

The MLR was calculated by dividing the monocyte count by the lymphocyte count, which were obtained directly from laboratory data files. The presence or absence of an MI diagnosis was our primary outcome variable.We determined MI status based on participants’ responses to the question within the miscellaneous pain questionnaire section, which inquired, “Has a doctor ever informed you that you had a heart attack (also known as myocardial infarction)?” Previous epidemiological studies (16–18) have effectively utilized self-reported MI measures, demonstrating their reliability.

The criteria for defining DM were established by the American Diabetes Association (19) and were determined using a self-report questionnaire. To be classified as having DM, participants were required to meet at least one of the following criteria (20): (1) Fasting plasma glucose (FPG) ≥ 126 mg/dl (7.0 mmol/L) (fasting defined as no caloric intake for at least 8 h); (2) 2-h post-glucose (PG) ≥ 200 mg/dl (11.1 mmol/L) during the oral glucose tolerance test (OGTT) conducted as per WHO guidelines, using a glucose load equivalent to 75 g of anhydrous glucose dissolved in water; (3) Hemoglobin A1c (HbA1C) ≥ 6.5% (48 mmol/mol), determined in a laboratory using an NGSP-certified and DCCT assay-standardized method; (4) self-reported questionnaire data confirming a physician's diagnosis of diabetes; and (5) reduced blood glucose due to the current use of insulin or diabetes medication.

Covariate data were collected through questionnaires, physical examinations, and laboratory tests. A range of potential covariates was assessed based on existing literature and clinical relevance. These covariates included age, sex, race/ethnicity, marital status, high blood pressure (HBP), body mass index (BMI), smoking status, physical activity, HbA1c, hemoglobin (HGB), high-sensitivity C-reactive protein (HSCRP), Vitamin D, high-density lipoprotein cholesterol (HDL), total cholesterol (TC), and duration of diabetes. Hypertension is characterized by a systolic blood pressure of at least 140 mmHg and a diastolic blood pressure of at least 90 mmHg, or by a previous diagnosis of hypertension (21). Smoking status was categorized in line with prior literature definitions as never smokers (those who had smoked fewer than 100 cigarettes) or current smokers (those who had smoked more than 100 cigarettes) (22). Physical activity was classified into three categories: sedentary, moderate, or vigorous. To fall under the moderate category, at least 10 min of activity per week was required, resulting in a slight increase in breathing or heart rate. For vigorous activity, at least 10 min of activity per week was required, resulting in a substantial increase in breathing or heart rate (23). The duration of diabetes was calculated by subtracting the age at which the subjects first learned of their diabetes from their reported screening age. Baseline laboratory tests included HbA1c, HGB, HSCRP, HDL, and TC. Dietary data provided information on participants’ 24-hour vitamin D intake. Detailed descriptions of all the variables mentioned above can be accessed on the NHANES website at https://wwwn.cdc.gov/Nchs/Nhanes/continuousnhanes.

### Statistical analysis

To assess the normal distribution of continuous variables, the Shapiro-Wilk test was employed, while differences in continuous and categorical variables were investigated using independent *t*-tests and chi-squared tests. Generalized additive models were employed to perform smooth curve fittings, enabling the assessment of both linear and non-linear relationships between MLR and MI while accounting for relevant covariate adjustments. MLR analysis was conducted separately for both the continuous and categorical variables (quartiles). The study utilized Logistic regression models were used to estimate the odds ratios (ORs) and their corresponding 95% confidence intervals (CIs) to assess the relationship between MLR and the occurrence of MI. Model 1 included adjustments for sociodemographic characteristics, such as age, gender, race, and marital status. Model 2 incorporated adjustments for HBP, BMI, smoking status, and physical exercise. Model 3 represented complete adjustments, encompassing the variables in Model 2 and HbA1c, HGB, HSCRP, Vitamin D, HDL, TC, and the duration of diabetes.

Furthermore, we conducted stratified analyses based on age (<60 years or ≥60 years), sex, HbA1C category (<6.5 or ≥6.5), HBP (yes or no), and smoking status (never, former, current). We assessed the importance of interactions by developing interaction terms between MLR and different subgroups, utilizing the Wald test for binary variables and the likelihood ratio test for multilevel variables. A forest plot was employed to depict the effects of various subgroups and the significance of interactions. To examine the robustness of the results, sensitivity analyses were carried out after excluding participants with extreme BMI values, i.e., BMI <18.5 kg/m^2^ and BMI >35 kg/m^2^. In order to assess the predictive value of MLR for MI, receiver operating characteristic (ROC) curves were plotted.

Given that the sample size was determined exclusively from existing data, there were no previous statistical power estimates. All statistical computations were executed utilizing software programs like R 3.3.2 (http://www.R-project.org, The R Foundation, Shanghai, China) and Free Statistics software version 1.8 (20). Descriptive statistics were applied to characterize all participants. A significance level of *p* < 0.05, determined through two-tailed testing, indicated statistical significance.

## Results

### Study population characteristics

Table 1 summarizes the demographic, socioeconomic, comorbidity, and baseline characteristics of the study populations. Among all the participants, 148 (11.4%) were identified as having experienced MI. According to the MLR quartiles, participants with the highest MLR values (Q4) were more likely to be male, married, previous smokers, sedentary, and without HBP. Statistically significant differences were observed in age, sex, race, smoking status, HBP, HbA1c, vitamin D intake, TC, and duration of diabetes among the four groups (all *P*-values < 0.05).

**Table 1:** Baseline characteristics of participants.

Variables	Total (*n* = 1,295)	Q1 (*n* = 313)	Q2 (*n* = 318)	Q3 (*n* = 333)	Q4 (*n* = 331)	*p*
Age(y)	62.2 ± 12.4	56.0 ± 12.7	60.9 ± 11.7	63.5 ± 12.0	67.8 ± 10.2	<0.001
Sex, *n* (%)						<0.001
Male	718 (55.4)	107 (34.2)	156 (49.1)	209 (62.8)	246 (74.3)	
Female	577 (44.6)	206 (65.8)	162 (50.9)	124 (37.2)	85 (25.7)	
Race/ethnicity, *n* (%)						<0.001
Non-Hispanic white	254 (19.6)	82 (26.2)	62 (19.5)	62 (18.6)	48 (14.5)	
Non-Hispanic black	393 (30.3)	53 (16.9)	67 (21.1)	117 (35.1)	156 (47.1)	
Mexican American	302 (23.3)	88 (28.1)	95 (29.9)	64 (19.2)	55 (16.6)	
Other	346 (26.7)	90 (28.8)	94 (29.6)	90 (27)	72 (21.8)	
Marriage, *n* (%)						0.104
Married	757 (58.5)	182 (58.1)	173 (54.4)	187 (56.2)	215 (65)	
Unmarried	118 (9.1)	34 (10.9)	32 (10.1)	29 (8.7)	23 (6.9)	
Other	420 (32.4)	97 (31)	113 (35.5)	117 (35.1)	93 (28.1)	
BMI (kg/m^2^)	32.5 ± 7.7	32.6 ± 6.9	32.5 ± 8.4	32.6 ± 7.8	32.5 ± 7.4	0.991
Smoking status, *n* (%)						<0.001
Current smokers	182 (14.1)	58 (18.5)	45 (14.2)	42 (12.6)	37 (11.2)	
Former smokers	465 (35.9)	71 (22.7)	117 (36.8)	123 (36.9)	154 (46.5)	
Never smokers	648 (50.0)	184 (58.8)	156 (49.1)	168 (50.5)	140 (42.3)	
physical exercise, *n* (%)						0.015
Vigorous	134 (10.3)	48 (15.3)	36 (11.3)	27 (8.1)	23 (6.9)	
Moderate	315 (24.3)	78 (24.9)	77 (24.2)	82 (24.6)	78 (23.6)	
Sedentary	846 (65.3)	187 (59.7)	205 (64.5)	224 (67.3)	230 (69.5)	
HBP, *n* (%)						<0.001
No	900 (69.5)	188 (60.1)	216 (67.9)	249 (74.8)	247 (74.6)	
Yes	395 (30.5)	125 (39.9)	102 (32.1)	84 (25.2)	84 (25.4)	
HbA1C, *n* (%)	7.5 ± 1.7	7.9 ± 2.0	7.5 ± 1.8	7.5 ± 1.6	7.1 ± 1.3	<0.001
HGB(g/dl)	13.8 (12.8, 14.8)	13.7 (12.8, 14.6)	13.7 (12.7, 14.7)	13.9 (12.9, 15.0)	13.8 (12.7, 14.9)	0.269
HSCRP	48.2 ± 15.0	48.1 ± 14.0	50.1 ± 16.7	47.8 ± 14.4	47.0 ± 14.5	0.051
Vit D (nmol/L)	176.6 ± 45.2	191.2 ± 50.3	181.9 ± 41.0	172.3 ± 42.9	162.2 ± 41.1	<0.001
HDL (mmol/L)	45.0 (38.0, 56.0)	47.0 (38.0, 56.0)	46.0 (39.0, 57.0)	45.0 (37.0, 56.0)	45.0 (37.0, 54.0)	0.153
TC (mmol/L)	176.6 ± 45.2	191.2 ± 50.3	181.9 ± 41.0	172.3 ± 42.9	162.2 ± 41.1	<0.001
Duration of diabetes(y)	11.0 (5.0, 18.0)	8.0 (4.0, 15.0)	10.0 (5.0, 18.0)	12.0 (5.0, 19.0)	13.0 (7.0, 20.0)	<0.001
MI, *n* (%)						<0.001
No	1,147 (88.6)	302 (96.5)	290 (91.2)	282 (84.7)	273 (82.5)	
Yes	148 (11.4)	11 (3.5)	28 (8.8)	51 (15.3)	58 (17.5)	

### Factors associated with MI

Univariate analysis showed that all covariates were associated with MI, and age, race/ethnicity, hypertension, former or current smoker, and duration of diabetes were positively associated with MI (Supplementary Table S1).

### Association between MLR and MI

**Figure 2:**
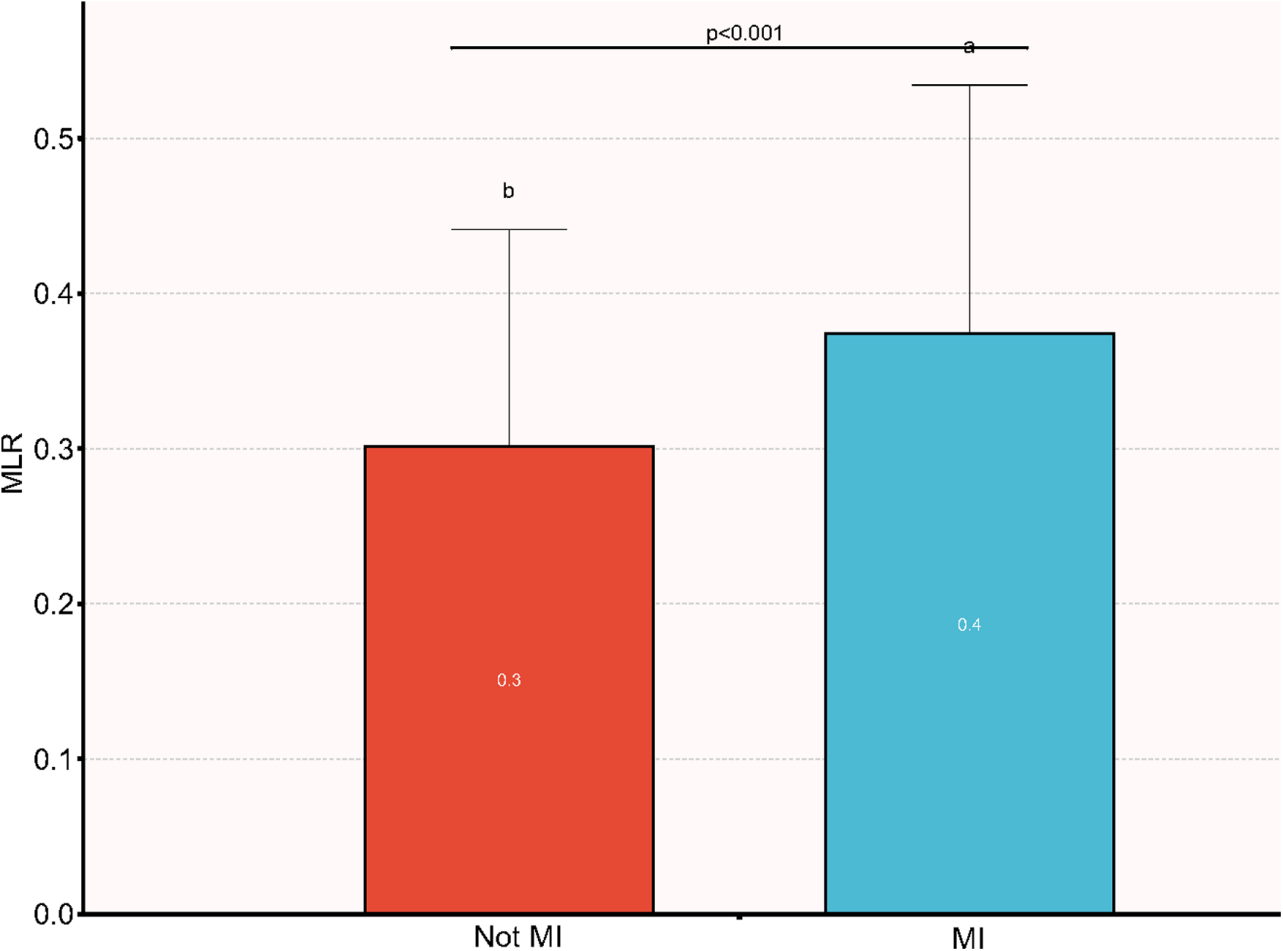
Comparison of MLR between patients with MI and non-MI. MI, myocardial infarction; MLR, monocyte-to-lymphocyte ratio.

The outcome of the analysis revealed that participants who had MI demonstrated a higher MLR than those who did not have MI, as depicted in Figure 2.The dissimilarities in MLR between the two groups are clearly illustrated in the figure (*p* < 0.001).

**Figure 3:**
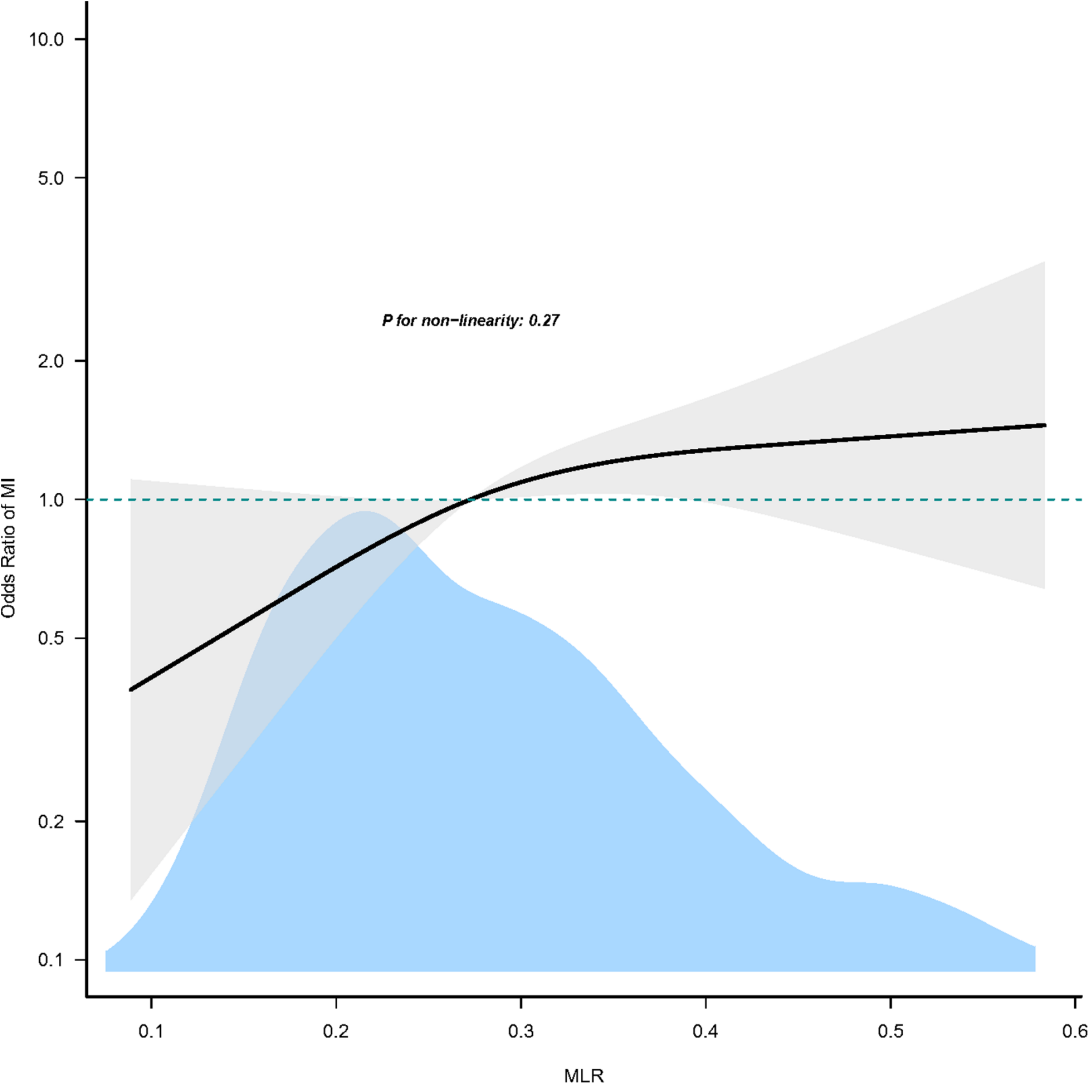
Association between MLR and MI. Only 95% of the data is shown. MI, myocardial infarction; MLR, monocyte-to-lymphocyte ratio.

Utilizing generalized additive models and smoothed curve fitting and following comprehensive adjustments for potential confounding variables, we established a linear relationship between MLR and MI in our study (p_for non−linearity_ = 0.27) (Figure 3).

Table 2 presents the odds ratios and their corresponding 95% confidence intervals (CIs) for the presence of MI concerning MLR. In the unadjusted model, MLR exhibited a statistically significant association with the presence of MI (OR = 1.32, 95% CI: 1.19∼1.46). Each 0.1 unit increase in MLR was linked to a 32% increase in the likelihood of MI. Subsequent multivariate regression models included stepwise adjustments. Model 1 incorporated age, gender, race/ethnicity, and marital status, resulting in an odds ratio of 1.16 (95% CI: 1.04∼1.31). Model 2 added HBP, BMI, smoking status, and physical exercise to the adjustments, yielding an odds ratio of 1.17 (95% CI: 1.04∼1.31). Finally, Model 3 introduced additional variables, namely HbA1c, HGB, HSCRP, vitamin D, HDL, TC, and diabetes duration, leading to an odds ratio of 1.14 (95% CI: 1.01∼1.29).

**Table 2:** Association between MLR and the presence of MI.

Quartiles	OR (95% CI)								
	NO.	Crude	*p*	Model 1	*p*	Model 2	*p*	Model 3	*p*
MLR*10		1.32 (1.19∼1.46)	<0.001	1.16 (1.04∼1.31)	0.009	1.17 (1.04∼1.31)	0.010	1.14 (1.01∼1.29)	0.041
Q1	313	1(Ref)		1(Ref)		1(Ref)		1(Ref)	
Q2	318	2.65 (1.30∼5.42)	0.008	2.01 (0.97∼4.16)	0.061	2.1 (1.01∼4.4)	0.048	2.13 (1.01∼4.47)	0.046
Q3	333	4.97 (2.54∼9.72)	<0.001	3.1 (1.55∼6.22)	0.001	3.02 (1.49∼6.1)	0.002	2.95 (1.45∼6)	0.003
Q4	331	5.83 (3.00∼11.34)	<0.001	2.96 (1.46∼5.98)	0.003	2.91 (1.42∼5.93)	0.003	2.74 (1.32∼5.69)	0.007
Trend test	1,295		<0.001		0.002		0.004		0.010

Since the values of MLR is a *10 (0 < a < 10). Carry out logistic regression analysis. When the independent variable is increased by 1 unit, the MLR value is equivalent to expanding by 10 times.

Furthermore, when MLR was treated as categorical variables, the associations with MI risk mirrored the trends observed in the continuous analyses. Participants were stratified into quartiles based on their 10-fold MLR values. In the unadjusted model, individuals in Q2, Q3, and Q4 exhibited a higher risk of MI compared to those in Q1 (the lowest quartile) (all *P* < 0.05). After comprehensive adjustments, the positive association between 10-fold MLR and MI risk remained. The odds ratios (ORs) were 2.13 (95% CI: 1.01∼4.47) in Q2, 2.95 (95% CI: 1.45∼6.00) in Q3, and 2.74 (95% CI: 1.32∼5.69) in Q4, relative to the Q1 group (all *P* < 0.05) (Table 2).

To evaluate the robustness of our results, we conducted a sensitivity analysis, excluding subjects with BMI <18.5 kg/m^2^ and BMI >35 kg/m^2^. The findings of this sensitivity analysis were consistent with those of the primary analysis, indicating that 10-fold MLR remained positively associated with MI, whether treated as a continuous or categorical variable (Table S2, all *P* < 0.05).

### Subgroup analyses outcomes

**Figure 4:**
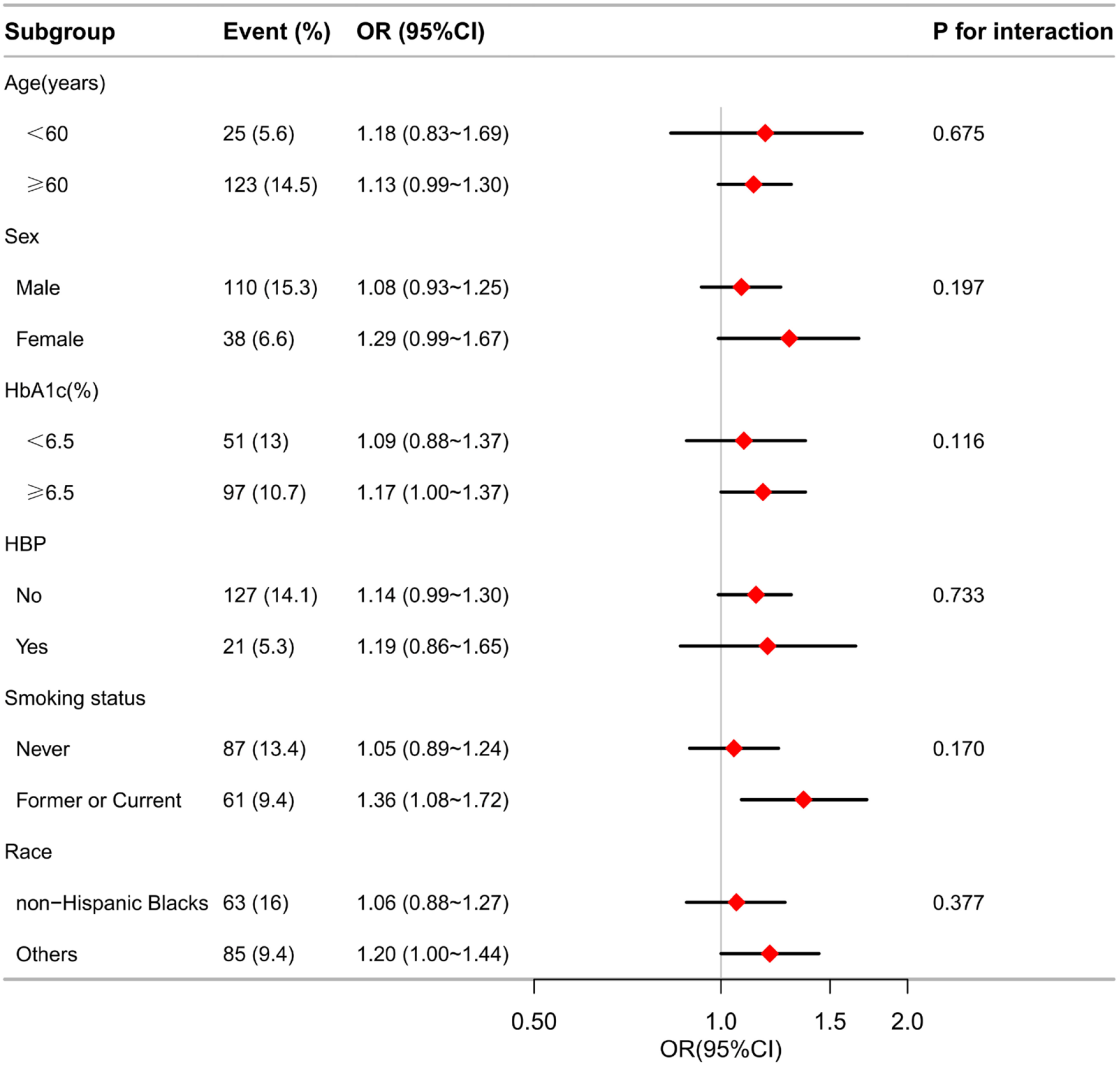
Effect size of MLR on the presence of MI in the age, sex, HbA1c, HBP, smoking status subgroup. OR, odds ratio; CI, confidence interval; MI, myocardial infarction; MLR, monocyte-lymphocyte ratio; HBP, hypertension; HbA1c, glycosylated hemoglobin.

The associations between MLR and MI risks were generally significant in multiple subgroups. In the subgroup analysis stratified by age, sex, HbA1C category (< 6.5 or ≥ 6.5), HBP (no or yes), and smoking status (never, former, or current), the association between MLR and the presence of MI is shown in Figure 4. The interaction analysis of MLR and age (p for interactio*n* = 0.545), MLR and sex (p for interaction = 0.112), MLR and HbA1c (p for interaction = 0.144), MLR and HBP (p for interaction = 0.687), and MLR and smoking status (p for interaction = 0.078) regarding the presence of PDR were not significant.

### Receiver operating characteristic curve analysis

We assessed the predictive value of MLR for MI, with an AUC (Area Under The Curve) of 0.661 (*P* < 0.05) (Supplemenatry Figure S1).The cut-off value of MLR was 0.103, and the sensitivity of MLR for predicting MI was 71.6% with a specificity of 56.8% (95% CI: 0.617∼0.706).

## Discussion

In this investigation, we harnessed the NHANES database as the cornerstone of our research. This study highlights a direct and significant association between increased MLR and the incidence of MI in the DM population. Remarkably, this relationship remained even after accounting for various confounding variables.

Recent investigations have illuminated the clinical relevance of MLR in various contexts. MLR, as a novel inflammatory marker, plays a pivotal role in the diagnosis and prognosis of conditions such as COVID-19 pneumonia (14, 24). Moreover, elevated MLR has been linked to an increased risk of severe limb ischemia and other vascular events in individuals with peripheral arterial occlusive disease (25). Similarly, MLR has been associated with adverse hospital outcomes in patients with ST-elevation myocardial infarction (STEMI) who undergo percutaneous coronary intervention (PCI) (26). Additional evidence highlights the independent association between high MLR and heightened risk of 6-month mortality in patients with acute myocardial infarction (AMI) treated with PCI (27). These studies collectively support the notion that combining monocyte and lymphocyte measurements, often obtained during routine clinical assessments, can serve as convenient and accurate prognostic indicators for various diseases.

Monocytes, a white blood cell circulating in the bloodstream and residing in specific tissues, become activated during immune and inflammatory responses (28). Their non-specific nature allows them to increase in response to various inflammatory conditions, with the ability to secrete critical cytokines such as tumor necrosis factor (TNF) and interleukin-1 (IL-1), both of which play indispensable roles in the inflammatory cascade (29). Monocytes also play a vital role in tissue repair and regeneration by migrating to damaged tissues, especially under conditions such as myocardial infarction and other injuries (30). On the other hand, lymphocytes contribute to endothelial cell proliferation and immune defense, and their levels may significantly decrease following trauma or injury (31). Such a decrease in lymphocyte count may be associated with apoptosis and immune cell dysfunction (32). Chronic inflammation has been consistently linked to an elevated risk of CVD, including MI and stroke (33–35). Lymphocytes, as central immune cells, are integral to inflammatory responses. Inflammation can stimulate lymphocyte activation and proliferation, resulting in increased lymphocyte count. A decrease in lymphocyte count may be associated with apoptosis and immune cell dysfunction (32). These observations suggest a potential association between MLR and cardiovascular disease, particularly MI.

MI is a prevalent and life-threatening clinical condition that is influenced by a multitude of factors. The inflammatory response plays a pivotal role in the pathogenesis of MI. White blood cells, widely used as clinical markers of inflammation, contribute to oxidative and proteolytic myocardial damage by releasing reactive oxygen species, proteases, leukotrienes, interleukins, and myeloperoxidase (36). At the molecular level, an elevated MLR, indicating a relative increase in monocytes, could imply a heightened inflammatory response (37). Monocytes, recognized for their involvement in the development of atherosclerotic plaques, traverse into the arterial wall. There, they transform into macrophages, playing a significant part in the advancement and precariousness of the plaque. These macrophages absorb lipids, evolving into foam cells, and release cytokines and proteolytic enzymes that break down the plaque's fibrous cap. This process can result in rupture and thrombus formation, potentially triggering a MI (38). In contrast, a decreased lymphocyte count, suggested by an elevated MLR, could reflect a weakened adaptive immune response and a limited ability to repair tissue after ischemic incidents. Lymphocytes, especially T cells, play an essential role in monitoring immune activity and regulating inflammation. A decrease in their population may result in an unbalanced resolution of inflammation and hindered removal of damaged cells, potentially facilitating the advancement of atherosclerosis and increasing the risk of MI (39). This also suggests a close correlation between elevated monocyte levels, extent of myocardial injury, and prognosis. The higher the monocyte count, the more severe the myocardial infarction, and the worse the prognosis. Lymphocytes, known for their role in orchestrating specific immune responses against infectious agents and foreign substances, are instrumental during inflammation.

Inflammatory processes can induce production of chemokines and adhesion molecules, leading to lymphocyte infiltration. Effector T cells are recruited to the myocardial infarction site, releasing proinflammatory cytokines and inducing an inflammatory response within the myocardium (40). Hence, lymphocytes are integral to the entire spectrum of the myocardial infarction process. Some studies have postulated that the increase in MLR may be linked to the production of proinflammatory chemokines such as interleukin-6 (IL-6), tumor necrosis factor, IL-1β, and monocyte chemoattractant protein-1. These chemokines significantly recruit and activate monocytes and leukocytes in MI patients, subsequently leading to inflammatory responses (41, 42). MLR, a newly discovered inflammatory marker, may offer a more stable reflection of the inflammatory response compared to independent levels of monocytes, lymphocytes, and leukocytes. This stability arises from the equilibrium between monocytes and lymphocytes, and is less influenced by various physiological and pathological conditions.

The findings of this study are clinically significant, suggesting that maintaining a low MLR in individuals with DM could be a crucial consideration in mitigating the risk of MI. MLR, as an easily accessible biomarker, may be valuable in clinical practice, enabling physicians to swiftly evaluate a patient's inflammatory status and cardiovascular risk. In patients with diabetes, those exhibiting higher levels of MLR may require more aggressive anti-inflammatory and cardiovascular protective strategies, including lifestyle modifications, medications, and enhanced monitoring (43, 44). Furthermore, MLR may assist in identifying individuals who could benefit from more intensive preventive measures, such as antiplatelet therapy and lipid-lowering interventions (45, 46). Our study leverages a vast and nationally representative sample of U.S. adults, characterized by sound demographics and relative homogeneity, enabling us to effectively control for confounding variables and their impact on the outcomes. Multiple potential confounders were adjusted, and sensitivity analyses corroborated our principal findings.

Nevertheless, certain limitations should be acknowledged. Firstly, our research hinges on data from the NHANES database, rendering it a cross-sectional study. While we established a robust association between MLR and MI, the cross-sectional design precludes any causal inference. Secondly, our study is centered exclusively on U.S. adults, potentially limiting the generalizability of our findings to other populations. Thirdly, we were unable to eliminate all potential residual confounders due to the presence of unmeasured confounders. Fourthly, the data on monocyte and lymphocyte counts were collected under fasting conditions, while the non-fasting data were not examined. Variations in laboratory tests may introduce bias. Finally, the use of self-reported myocardial infarction (MI) could have skewed our findings due to potential subjectivity. In light of these limitations, it is essential to design a multicenter controlled trial to validate our results in future studies.

## Conclusions

Our study establishes a crucial connection between MLR and the risk of myocardial infarction in diabetic patients, highlighting the potential of MLR as a significant predictor of cardiovascular risk in this population. This finding has important implications for risk stratification and management strategies for diabetic patients.

## Data Availability

Publicly available datasets were analyzed in this study. This data can be found here: www.cdc.gov/nchs/nhanes/.
