# Supplementary material for: Association of monocyte-lymphocyte ratio and myocardial infarction in the U.S. population with diabetes
Source: Front Cardiovasc Med. 2024 Sep 27;11:1432838. doi: 10.3389/fcvm.2024.1432838 (PMC11472363; doi:10.3389/fcvm.2024.1432838)
Supplement: Supplementary file 1 [file Datasheet1.docx]

**Table S1.** Univariate analysis for the presence of MI.

| **Variable** | **OR_95CI** | **P** |
| --- | --- | --- |
| **Age** | 1.05 (1.03~1.07) | <0.001 |
| **Sex** |  |  |
| Male | 1 |  |
| Female | 0.39 (0.26~0.57) | <0.001 |
| **Race/ethnicity, n (%)** |  |  |
| Non-Hispanic white | 1 |  |
| Non-Hispanic black | 2.5 (1.44~4.34) | 0.001 |
| Mexican American | 1.5 (0.82~2.75) | 0.19 |
| Other | 1.52 (0.84~2.75) | 0.163 |
| **Marriage, n (%)** |  |  |
| Married | 1 |  |
| Unmarried | 0.41 (0.18~0.97) | 0.041 |
| Other | 1.16 (0.81~1.66) | 0.419 |
| **BMI** | 1 (0.98~1.02) | 0.921 |
| **Smoking status, n (%)** |  |  |
| Current smokers | 1 |  |
| Former smokers | 0.91 (0.55~1.49) | 0.696 |
| Never smokers | 0.62 (0.38~1.02) | 0.06 |
| **physical exercise, n (%)** |  |  |
| Vigorous | 1 |  |
| Moderate | 1.58 (0.78~3.19) | 0.202 |
| Sedentary | 1.46 (0.76~2.81) | 0.251 |
| **HBP, n (%)** |  |  |
| No | 1 |  |
| Yes | 2.93 (1.81~4.72) | <0.001 |
| **HbA1C, n (%)** |  |  |
| <6.5 | 1 |  |
| >=6.5 | 0.8 (0.56~1.15) | 0.23 |
| **HGB** | 0.99 (0.89~1.1) | 0.8 |
| **HSCRP** | 1.01 (0.99~1.02) | 0.23 |
| **Vit D** | 0.99 (0.95~1.02) | 0.513 |
| **HDL** | 0.98 (0.96~0.99) | 0.001 |
| **TC** | 0.99 (0.99~1) | <0.001 |
| **Duration Of Diabetes(y)** | 1.03 (1.01~1.04) | <0.001 |

**Table S2**. Sensitivity analyses investigating associations between MLR and MI after excluding participants who with extreme BMI (18.5kg/m^2^≤BMI ≤35kg/m^2^, 899).

|  | **MI (n=107)** | **OR (95% CI)** | | | | | | |
| --- | --- | --- | --- | --- | --- | --- | --- | --- |
|  | **Crude** | **p** | **Model1** | **p** | **Model2** | **p** | **Model3** | **p** |
| MLR*10 | 1.32 (1.18~1.47) | <0.001 | 1.19 (1.05~1.35) | 0.006 | 1.19 (1.05~1.36) | 0.009 | 1.18 (1.02~1.35) | 0.024 |
| MLR*10 quartiles |  |  |  |  |  |  |  |  |
| Q1 | 1(Ref) |  | 1(Ref) |  | 1(Ref) |  | 1(Ref) |  |
| Q2 | 2.22 (0.98~5.03) | 0.055 | 1.77 (0.77~4.07) | 0.176 | 1.87 (0.80~4.33) | 0.146 | 1.85 (0.79~4.33) | 0.157 |
| Q3 | 4.44 (2.08~9.48) | <0.001 | 2.83 (1.29~6.21) | 0.010 | 2.75 (1.24~6.11) | 0.013 | 2.72 (1.21~6.11) | 0.015 |
| Q4 | 5.80 (2.76~12.2) | <0.001 | 3.11 (1.41~6.88) | 0.005 | 3.11 (1.40~6.92) | 0.005 | 3.02 (1.32~6.88) | 0.009 |
| Trend test |  | <0.001 |  | 0.002 |  | 0.003 |  | 0.006 |

Since the values of MLR is a*10(0 < a < 10). Carry out logistic regression analysis. When the independent variable is increased by 1 unit, the MLR value is equivalent to expanding by 10 times.


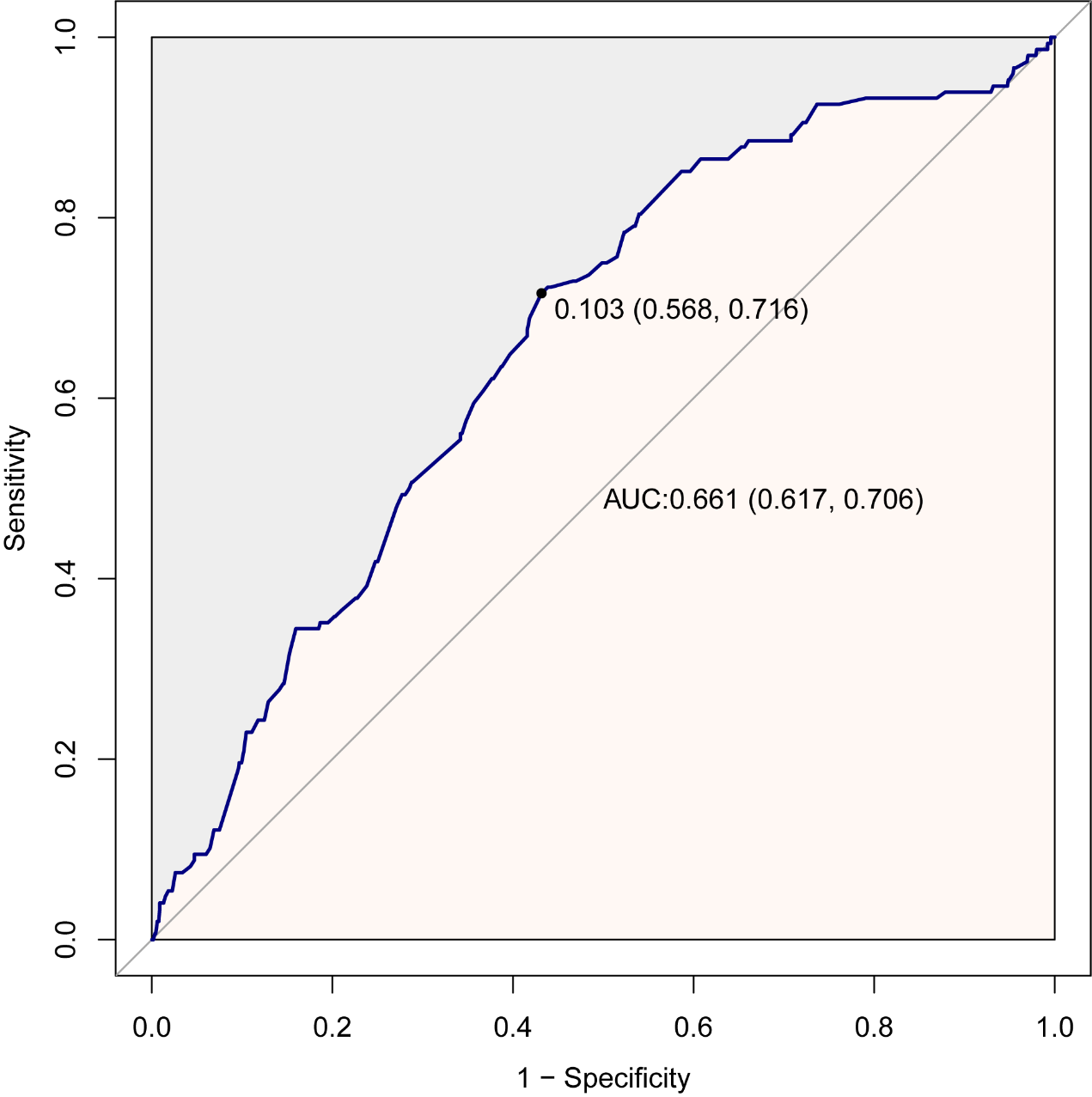


**Figure S1.** The receiver operating characteristic (ROC) curve.
